# Unique niche-specific adaptation of fructophilic lactic acid bacteria and proposal of three *Apilactobacillus* species as novel members of the group

**DOI:** 10.1186/s12866-021-02101-9

**Published:** 2021-02-09

**Authors:** Shintaro Maeno, Hiroya Nishimura, Yasuhiro Tanizawa, Leon Dicks, Masanori Arita, Akihito Endo

**Affiliations:** 1 Department of Food, Aroma and Cosmetic Chemistry, Tokyo University of Agriculture, Abashiri, Hokkaido 099-2493 Japan; 2 Department of Informatics, National Institute of Genetics, Mishima, Shizuoka, 411-8540 Japan; 3 Department of Microbiology, University of Stellenbosch, Matieland, Stellenbosch, 7602 South Africa; 4 RIKEN Center for Sustainable Resource Science, Yokohama, Kanagawa 230-0045 Japan

**Keywords:** Fructophilic lactic acid bacteria, Convergent evolution, Comparative genomics, *Apilactobacillus*, *Fructobacillus*, *adhE*

## Abstract

**Background:**

Fructophilic lactic acid bacteria (FLAB) found in D-fructose rich niches prefer D-fructose over D-glucose as a growth substrate. They need electron acceptors for growth on D-glucose. The organisms share carbohydrate metabolic properties. *Fructobacillus* spp., *Apilactobacillus kunkeei,* and *Apilactobacillus apinorum* are members of this unique group. Here we studied the fructophilic characteristics of recently described species *Apilactobacillus micheneri*, *Apilactobacillus quenuiae,* and *Apilactobacillus timberlakei*.

**Results:**

The three species prefer D-fructose over D-glucose and only metabolize D-glucose in the presence of electron acceptors. The genomic characteristics of the three species, i.e. small genomes and thus a low number of coding DNA sequences, few genes involved in carbohydrate transport and metabolism, and partial deletion of *adhE* gene, are characteristic of FLAB. The three species thus are novel members of FLAB. Reduction of genes involved in carbohydrate transport and metabolism in accordance with reduction of genome size were the common characteristics of the family *Lactobacillaceae,* but FLAB markedly reduced the gene numbers more than other species in the family. Pan-genome analysis of genes involved in metabolism displayed a lack of specific carbohydrate metabolic pathways in FLAB, leading to a unique cluster separation.

**Conclusions:**

The present study expanded FLAB group. Fructose-rich environments have induced similar evolution in phylogenetically distant FLAB species. These are examples of convergent evolution of LAB.

**Supplementary Information:**

The online version contains supplementary material available at 10.1186/s12866-021-02101-9.

## Introduction

Fructophilic lactic acid bacteria (FLAB) are found in D-fructose-rich niches, such as flowers, fruits, fermented fruits, and the gastrointestinal tract of insects [1–3]. They actively metabolize D-fructose but show very limited growth on D-glucose as a growth substrate. External electron acceptors markedly improve their growth on D-glucose [2, 4], and D-Fructose, pyruvate, and O_2_ are used as electron acceptors. FLAB metabolically belong to a group of heterofermentative lactic acid bacteria (LAB), but profiles of end-products from the metabolism of D-glucose clearly differentiate FLAB from other heterofermentative LAB. Lactate, ethanol, and CO_2_ are the major end-products in the heterofermentative pathway of LAB. In the case of FLAB, ethanol is replaced with acetate [2, 4]. These phenotypic characteristics place FLAB in an unusual entity within the LAB group that generally prefers D-glucose and anaerobic conditions for growth. Recent studies showed that the unique growth characteristics in FLAB are due to complete or partial deletion of the *adhE* gene that encodes for a bifunctional alcohol/acetaldehyde dehydrogenase, AdhE [5–7]. In heterofermentative LAB, the AdhE enzyme in the phosphoketolase pathway plays a key role in maintaining the NAD/NADH balance during D-glucose metabolism [8]. In FLAB, external electron acceptors oxidize NADH to NAD and keep the NAD and NADH in balance. Introduction of *adhE* in FLAB markedly changed their metabolic properties, and the introduction enabled them to metabolize D-glucose in the absence of the electron acceptors [7]. Moreover, FLAB are poor carbohydrate fermenters and metabolize only a limited number of carbohydrates [9].

*Fructobacillus* spp. are representatives of the FLAB group and fructophilic characteristics are well conserved in all species of the genus [4]. *Lactobacillus kunkeei* and *Lactobacillus apinorum,* recently reclassified as *Apilactobacillus kunkeei* and *Apilactobacillus apinorum*, respectively [10], are also members of FLAB [11]. Although these two groups are phylogenetically distant, they share similar genomic characteristics, i.e. small genomes (< 1.69 Mbp) and thus a low number of coding DNA sequences (CDSs) [5, 12]. Fewer genes are involved in carbohydrate transport and metabolism, and complete phosphotransferase system (PTS) transporters are lacking in FLAB [5, 12]. These genetic characteristics agree with their poor carbohydrate metabolic properties [1, 13]. The genomic characteristics suggest that FLAB underwent reductive evolution to adapt to fructose-rich niches. A recent study reported a pseudo-FLAB strain belonging to *Leuconostoc citreum* from satsuma mandarin peel [14]. The strain showed similar growth characteristics to FLAB, i.e. minor growth on D-glucose, active growth on D-fructose, and improved growth by supplementation of the electron acceptors on D-glucose. The growth characteristics was due to an inactivation of the *adhE* gene by deletion of the promoter region upstream of the gene [14]. However, the strain did not have specific reduction of total CDS and genes involved in carbohydrate transport and metabolism, observed in typical FLAB, and the strain was thus described as pseudo-FLAB.

*Apilactobacillus kunkeei* and *A. apinorum* have been isolated from flowers and/or honey bee guts, and share high 16S rRNA gene similarities (> 99%) [3, 15]. In a recent study, three novel species, *Lactobacillus micheneri, Lactobacillus quenuiae*, and *Lactobacillus timberlakei,* were isolated from flowers and guts of wild bees [16]. These species share relatively high 16S rRNA gene sequence similarities (97%) with *A. kunkeei* and *A. apinorum* and have been reclassified as members of the genus *Apilactobacillus* [10]. The three novel species were routinely cultured in broth supplemented with D-fructose and fermented only a few carbohydrates [16], implicating that they are novel members in the FLAB group. Despite a great interest to understand niche-specific adaptation of LAB, fructophilic characteristics of the three species have, thus far, not been described.

In the present study, the biochemical and genomic characteristics of *Apilactobacillus micheneri*, *Apilactobacillus timberlakei,* and *Apilactobacillus quenuiae* were studied. Gene components in the genomes of FLAB were compared with those of LAB. The study clearly indicated a unique evolutional trend in FLAB and provide evidence that fructose-rich niches have induced similar gene reductions in phylogenetically distant LAB.

## Results

### Growth and metabolic characteristics

**Fig. 1:**
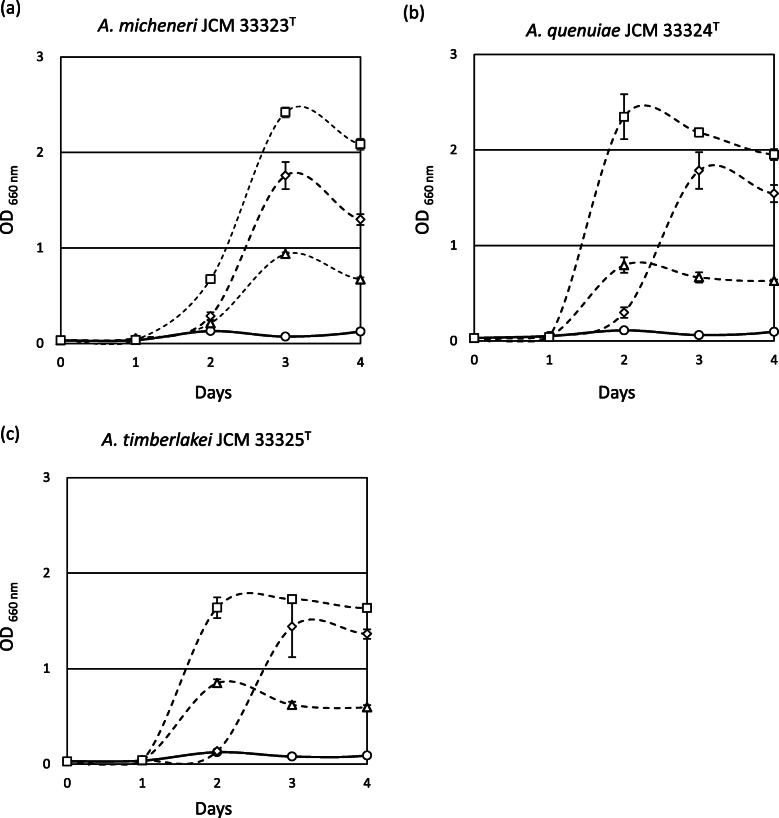
Growth characteristics of *A. micheneri* JCM 33323^T^ (**a**), *A. quenuiae* JCM 33324^T^ (**b**), and *A. timberlakei* JCM 33325^T^ (**c**) in GYP broth (O), FYP broth (Δ), GYP-P broth (◇), and GYP broth under aerobic conditions (□). Data indicate means ± standard deviations (error bars)

*Apilactobacillus micheneri, A. timberlakei,* and *A. quenuiae* grew well in D-fructose-yeast extract-peptone (FYP) broth, but poorly in D-glucose-yeast extract-peptone (GYP) broth (Fig. 1). However, supplementation of pyruvate (GYP-P) and aerobic culturing (GYP-O_2_) markedly enhanced their growth on D-glucose. The three species produced mainly lactate and acetate, with trace amounts of ethanol, from the metabolism of D-glucose (Table 1). Mannitol was produced at a level of 0.44 mM by the strains during growth in GYP broth supplemented with D-fructose. Carbohydrates were poorly fermented, with only two of the 49 carbohydrates in API50CHL galleries showing a positive reaction. A weak reaction was recorded from only one carbohydrate (glucose) by *A. timberlakei*. The three species fermented D-fructose within 3 to 4 days but took 5 days to ferment D-glucose, except for *A. timberlakei* that produced a weak reaction from the metabolism of D-glucose. Of the three species, only *A. timberlakei* metabolized sucrose after 5 days. The unique growth and metabolic properties recorded for these species are consistent with those of FLAB, i.e. *A. kunkeei, A. apinorum,* and *Fructobacillus* spp.

**Table 1:** End products from D-glucose

Strain	Consumed D-glucose (mM)	End products (mM)^a^			Molar ratio of L:E:A	Carbon recovery (%)
		L	E	A		
*A. micheneri* JCM 33323^T^	3.84 (±0.66)^b^	4.49 (±0.94)	0.04 (±0.19)	2.38 (±0.08)	1:0.01:0.53	79.5
*A. quenuiae* JCM 33324^T^	4.46 (±0.44)	5.50 (±0.84)	0.03 (±0.08)	2.95 (±0.23)	1:0.01:0.54	84.0
*A. timberlakei* JCM 33325^T^	3.55 (±0.86)	4.48 (±0.95)	0.02 (±0.05)	2.24 (±0.01)	1:0.01:0.50	84.3

^a^*L* lactate, *E* ethanol, *A* acetate

^b^Mean (± standard deviation)

### Genomic features of *A. micheneri*,  *A. quenuiae*, and *A. timberlakei*

The genome sizes of *A. micheneri, A. timberlakei,* and *A. quenuiae* were 1.46, 1.58, and 1.54 Mbp and the number of CDSs 1485, 1553, and 1570, respectively. These were similar to the genome sizes and number of CDSs reported for known FLAB (Table 2, Supplemental Table S1). The gene content profiles of the three species, based on 21 functional categories of Cluster of Orthologous Groups (COG), are summarized in Table 3. The gene content profiles of the three species were similar and shared similarity to those of known FLAB. All three species have only 53 to 55 genes involved in carbohydrate transport and metabolism (class G in COG) and the ratio of these genes against total number of genes in all COGs were only 3.4 to 3.6%, with values markedly lower than recorded for other *Lactobacillaceae* species (median ± SD, 8.5 ± 2.4%). These findings are consistent with the unique characteristics of known FLAB [9]. Class G ranked 9th or 10th among the 21 COG categories. Slight differences in amino acid transport and metabolism (class E) were recorded between the three species and *A. kunkeei* and *A. apinorum*. The ratio of class E in *A. micheneri, A. timberlakei,* and *A. quenuiae* were 5.3 to 5.7% and the class ranked the 5th or 6th largest, whereas class E in *A. kunkeei* and *A. apinorum* were 7.1 to 8.4% and ranked 2nd. A similar profile was also observed in *Apilactobacillus ozensis*, which does not have fructophilic growth characteristics [17]. In agreement with the small number of class G genes, *Apilactobacillus micheneri, A. timberlakei,* and *A. quenuiae* possessed only one PTS gene (Table 4), and no complete PTS transporters. Most genes used for TCA cycle and ubiquinone and other terpenoid-quinone biosynthesis pathway were not found, although aerobic culturing markedly enhanced growth on D-glucose.

**Table 2:**
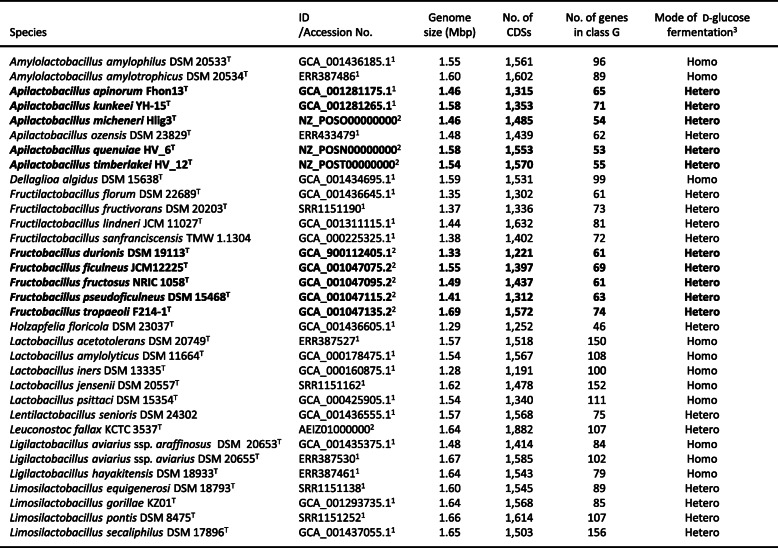
Species possessing small genomes (< 1.69 Mbp) included in the present study

^1^Data from DAGA

^2^Data from NCBI Database

^3^Mode of D- glucose fermentation (Homo homofermentative, Hetero heterofermentative

**Table 3:**
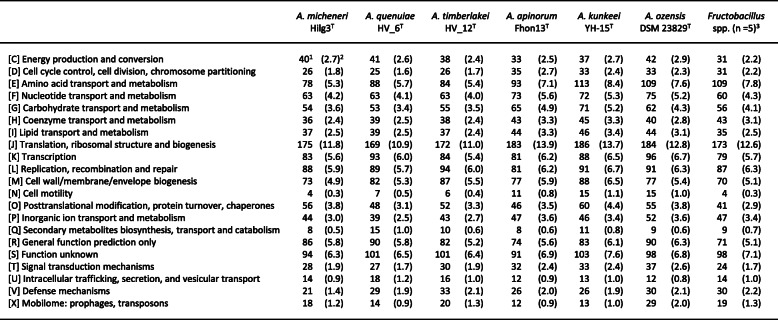
Gene content profiles obtained for *Apilactobacillus* spp. and *Fructobacillus* spp

^1^Number of genes in each COG class

^2^Ratio of genes against the total number of genes in all COGs

^3^The values for Fructobacillus spp. indicate means (standard deviation) of the number of genes used for the pathways in all five species

**Table 4:**
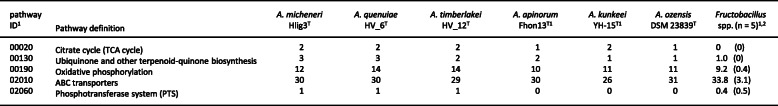
Number of genes in each metabolic pathway found in *Apilactobacillus* spp. and *Fructobacillus* spp

^1^Data for A. apinorum, A. kunkeei, and Fructobacillus spp. were obtain from elsewhere (12)

^2^2The values for Fructobacillus spp. indicate means (standard deviation) of the number of genes used for the pathways in all five species

The metabolic and genomic characteristics of *A. micheneri, A. quenuiae,* and *A. timberlakei* observed in this study are consistent with those of known FLAB, and the three species are considered as members of the FLAB group.

**Fig. 2:**
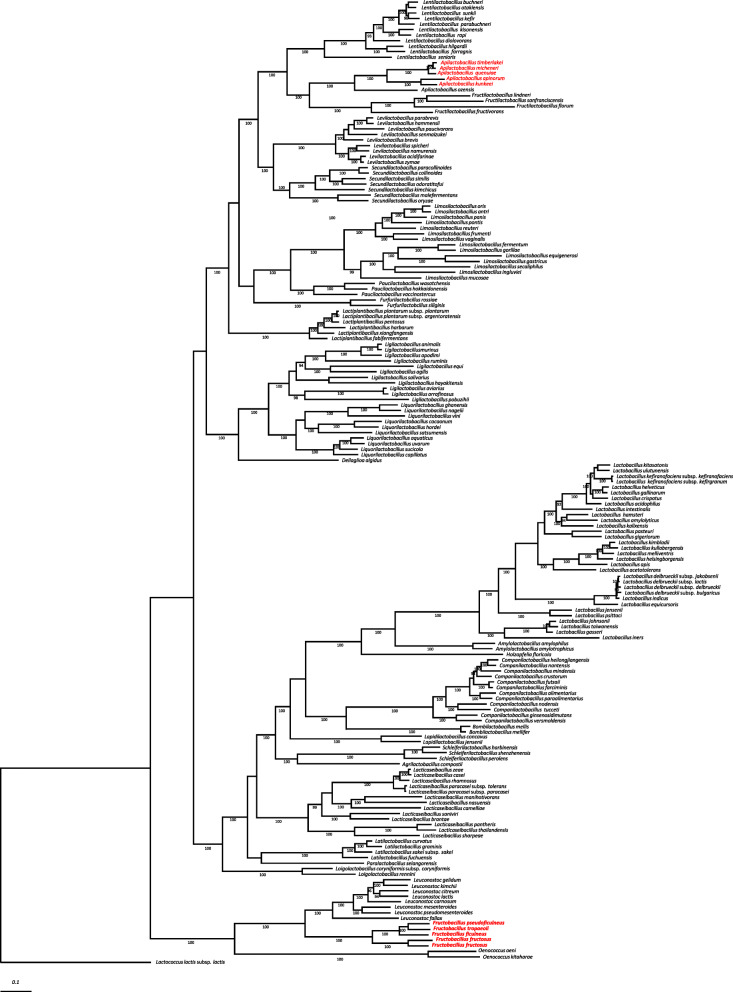
Core genome phylogenetic tree of FLAB (shown with red letter) and other *Lactobacillaceae* species based on the multiple alignment of protein sequences for the 87 single-copy genes. The maximum-likelihood tree was constructed using the best-fit evolutionary model using the 174 strains in the family *Lactobacillaceae*. The values on the branches are bootstrap support from 1000 rapid bootstrapping replicates, and only values over 90% were indicated. *Lactococcus lactis* IL1403 (NC_002662.1) was used as an out group

A core genome phylogenetic tree was produced based on concatenated sequences of 87 single-copy core genes to confirm the phylogenetic relationships between FLAB species. The tree clearly separated FLAB species into two phylogenetic groups, i.e. the genera *Apilactobacillus* and *Fructobacillus* (Fig. 2). *Apilactobacillus* spp. were phylogenetically related to *Fructilactobacillus* spp., and *Fructobacillus* spp. produced a cluster with members of the former family *Leuconostocaceae,* i.e. *Leuconostoc* spp. and *Oenococcus* spp.

### Evolution of FLAB species

**Fig. 3:**
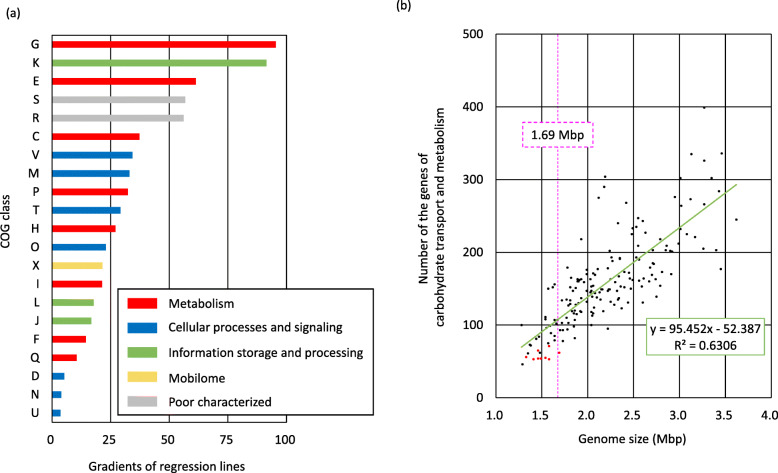
Correlation between genome sizes and number of genes assigned in each COG class of 174 strains in the family *Lactobacillaceae*. **a** shows gradients of regression lines of each COG class and (**b**) shows correlation between genome size and number of genes involved in carbohydrate transport and metabolism (class G) in the 174 strains. Function of each COG class is shown in Table 3. FLAB species were marked with red points in (**b**)

To study the evolutional trend of FLAB in the family *Lactobacillaceae,* genomes of 174 strains (167 species and 11 subspecies) were analyzed. The CDSs of the 174 strains of the family *Lactobacillaceae,* including ten strains of FLAB species, were categorized into 21 COG classes based on their functions. The numbers of genes assigned in the classes were compared with genome sizes, and gradients of regression lines were obtained for each class. The steepest gradient was obtained in class G (carbohydrate transport and metabolism; slope = 95.5, r^2^ = 0.64), followed by class K (transcription; gradient = 91.4, r^2^ = 0.82) (Fig. 3a and Supplemental Fig. S1). FLAB species, i.e. *Fructobacillus* spp. and *Apilactobacillus* spp. (excluding *A. ozensis*), were placed well below the regression line in class G (Fig. 3b), whereas in class K, they were close to the regression line (Supplemental Fig. S1). Class E (amino acid transport and metabolism), class S (function unknown), and class R (general function prediction only) showed similar gradient values, i.e. 61.3 (r^2^ = 0.54), 56.8 (r^2^ = 0.71), and 56.1 (r^2^ = 0.84), respectively. Even in classes related to similar functions (e.g. metabolism), gradients differed markedly (Fig. 3a).

### Pan-genome analysis based on genes involved in metabolism

**Fig. 4:**
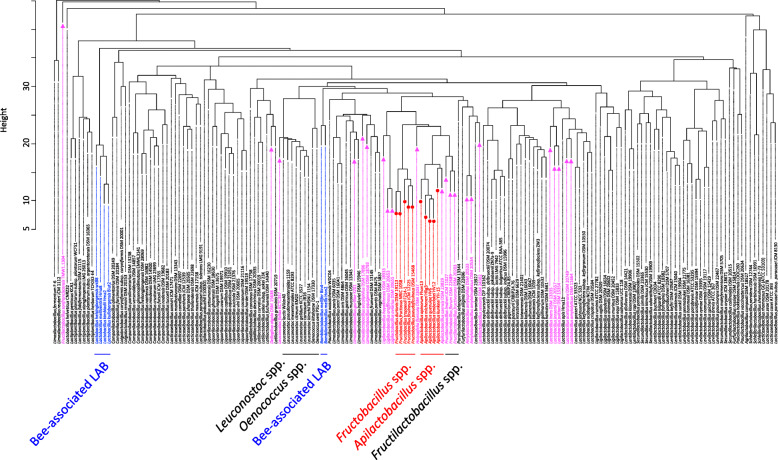
The dendrogram shows hierarchical clustering produced based on presence/absence of genes involved in metabolism. FLAB were marked in red, species possessing small genomes (< 1.69 Mb) in pink, and bee-associated LAB in blue

To characterize gene profiles involved in metabolism of FLAB, genes involved in metabolic pathways were predicted in the 174 (sub) species of the family *Lactobacillaceae,* and a dendrogram was produced based on presence/absence of the genes. Species containing small genomes (< 1.69 Mbp) received special attention to focus on relationships between genomic size and observed genes for metabolism. Several clusters were produced in the dendrogram and the separation of clusters was generally in agreement with the taxonomic positions in the family (Fig. 4). A few exceptions were noted, e.g. *Lactobacillus* spp. and *Limosilactobacillus* spp. were each separated into three groups. *Apilactobacillus micheneri, A. quenuiae*, and *A. timberlakei* clustered with *A*. *apinorum, A. kunkeei,* and *Fructobacillus* spp*.* (Fig. 4). *Apilactobacillus ozensis*, *Fructilactobacillus* spp., and *Holzapfelia floricola* were also included in the cluster. The cluster was composed of species containing small genomes (< 1.69 Mbp). *Leuconostoc* spp. and *Oenococcus* spp., phylogenetic relatives of *Fructobacillus* spp., positioned distantly from *Fructobacillus* spp. and formed a single cluster. Species possessing small genomes (< 1.69 Mbp) spread into several clusters. Several FLAB species are associated with bees. However, other bee-associated species, i.e. five species in the *Lactobacillus helsingborgensis*-group and two species in the *Bombilactobacillus mellifer-mellis-*group, referred to as “Firm-5” and “Firm-4”, respectively [18], positioned distantly from FLAB.

A search for ‘specific’ genes present in more than 80% of FLAB species and less than 20% of other species in *Lactobacillaceae* (*n* = 164) yielded only one gene, encoding the K06872 uncharacterized protein (Table 5). BLAST analysis revealed that the protein is a TPM domain-containing protein, mainly conserved in *Apilactobacillus* spp., *Fructobacillus* spp., and *Lacticaseibacillus* spp. Likewise, the search for FLAB ‘missing’ genes yielded 16 genes (Table 5). These included genes involved in carbohydrate transport and metabolism, encoding IIB, IIC, and IID components of a mannose-specific PTS system, N-acetylglucosamine-6-phosphate deacetylase [EC:3.5.1.25], β-phosphoglucomutase [EC:5.4.2.6], glucosamine-6-phosphate deaminase [EC:3.5.99.6], UDP-glucose-hexose-1-phosphate uridylyltransferase [EC:2.7.7.12], galactokinase [EC:2.7.1.6], aldose 1-epimerase [EC:5.1.3.3], and maltose phosphorylase [EC:2.4.1.8]. Genes encoding inorganic cation transport (Cd^2+^/Zn^2+^-exporting ATPase [EC:3.6.3.3 3.6.3.5]), lipid metabolism (myosin cross-reactive antigen), and stress response proteins (osmoprotectant transport system ATP-binding protein and heat shock protein HtpX [EC:3.4.24.-]) were also found. When *A. ozensis,* sharing similar genetic characteristics to FLAB, was added to the FLAB group (*Fructobacillus-Apilactobacillus* group, *n* = 11), an additional gene was added to the one ‘specific’ gene and four genes to the 16 ‘missing’ genes (Table 5). The four ‘missing’ genes included a gene encoding mannose-6-phosphate isomerase [EC:5.3.1.8].

**Table 5:**
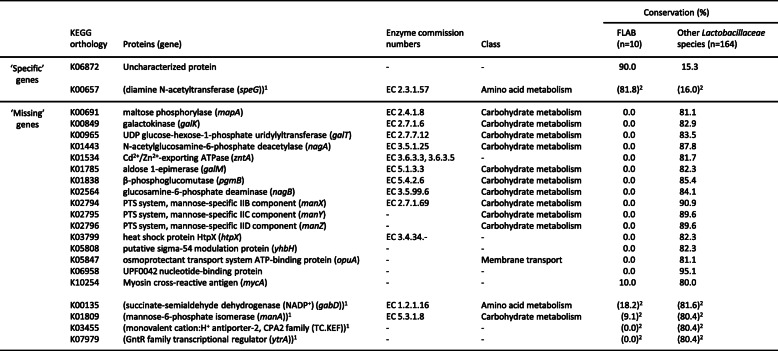
‘Specific’ and ‘missing’ genes found in FLAB. ‘Specific’ genes and ‘missing’ genes in FLAB were defined as genes conserved in greater than 80% of FLAB species and less than 20% of other species in *Lactobacillaceae*, and genes conserved in less than 20% of FLAB species and greater than 80% of other species in *Lactobacillaceae*, respectively

^1^Protein names shown in parenthesis were further added as ‘specific’ or ‘missing’ proteins in *Fructobacillus-Apilactobacillus* group (*n* = 11) to the single specific and 16 ‘missing’ genes found in FLAB, when *A. ozensis* was included in this group

^2^Conservation ratio was calculated for *Fructobacillus-Apilactobacillus* group and other Lactobacillaceae species, when A. ozensis was included in the former group

### Characterization of the *adhE* gene and relevant enzyme activities in FLAB

**Fig. 5:**
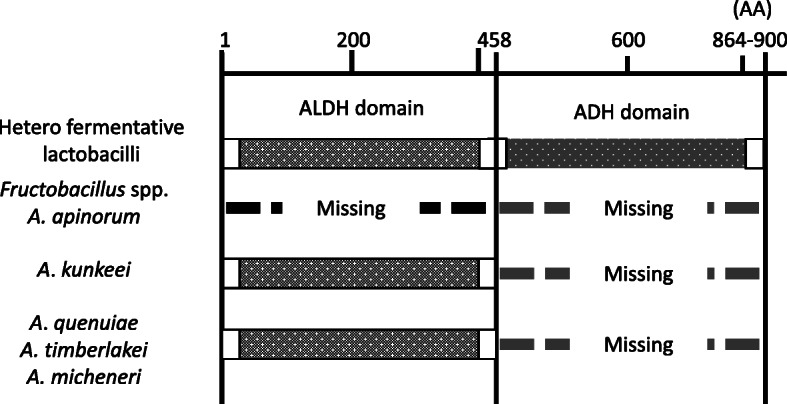
Primary structures of AdhE in FLAB

Heterofermentative LAB usually possess a single *adhE* gene, encoding a 864–900 amino acids AdhE protein containing both alcohol dehydrogenase (ADH) and acetaldehyde dehydrogenase (ALDH) domains. Since the lack of activities in ALDH and ADH has a significant impact on the growth of FLAB, the *adhE* gene was screened for presence in the genomes of *A. micheneri, A. quenuiae*, and *A. timberlakei*. Genome analysis revealed that the three species possessed a single *adhE* gene (1377 bp), encoding 458 amino acids that contained only the ALDH domain and not the ADH domain (Fig. 5). This genetic characteristic is consistent with *A. kunkeei* (Fig. 5). *Apilactobacillus apinorum* completely lacks *adhE* [11], but *A. ozensis* possess two *adhE* genes of different length, encoding 458 and 877 amino acids [5]. The sequence of AdhE in *A. micheneri*, *A. quenuiae,* and *A. timberlakei* shared a 94.5 to 95.4% similarity. The sequence similarities of the three species were 75.5 to 76.8% related to AdhE of *A. kunkeei* and 73.7 to 75.6% related to the shorter AdhE (one with 458 amino acids) in *A. ozensis*.

*Apilactobacillus quenuiae* showed relatively strong ALDH activity, whereas *A. micheneri* and *A. timberlakei* displayed weak or no ALDH activity (Table 6). None of the three species displayed ADH activity. Strong NADH oxidase activities were observed in all three species.

**Table 6:** Enzyme activities of ADH, ALDH, and NADH oxidase

Strain	ADH	ALDH	NADH oxidase
*A. micheneri* JCM 33323^T^	0	> 1 (± 0)^a^	2411 (±713)
*A. quenuiae* JCM 33324^T^	0	89 (±41)	478 (±343)
*A. timberlakei* JCM 33325^T^	0	0	1310 (± 57)
			(mU/mg protein)

^a^Mean (± standard deviation)

## Discussion

LAB have adapted to diverse environments at genomic level, resulting in diverse phenotypic characteristics according to their habitats [19, 20]. Of the genomic level adaptation characterized in LAB, the adaptation of FLAB to fructose-rich niches is one of the best-characterized and marked adaptations. The genomic characteristics of FLAB clearly separate the group from phylogenetic relatives [5, 12].

Novel bee-commensals *A. micheneri*, *A. quenuiae,* and *A. timberlakei* showed typical fructophilic characteristics, i.e. active growth on D-fructose, poor growth on D-glucose, enhanced growth on D-glucose in the presence of electron acceptors such as pyruvate and O_2_, acetate production instead of ethanol from D-glucose metabolism, mannitol production from D-fructose, poor carbohydrate metabolic properties, and lack of ADH activity (Fig. 1, Table 1). Genomic characteristics of *A. micheneri*, *A. quenuiae,* and *A. timberlakei*, including small genome size, small number of CDSs and genes involved in carbohydrate transport and metabolism, lack of complete PTS transporters, and partial deletion of the *adhE* gene (Tables 2, 3, and 4 and Fig. 5), were consistent with findings reported for FLAB. These characteristics clearly classify *A. micheneri*, *A. quenuiae,* and *A. timberlakei* as novel members of FLAB. *Apilactobacillus ozensis*, isolated from flowers, ferments D-glucose actively and produce ethanol [17]. Growth is repressed when cultured aerobic. Only D-glucose and D-fructose are metabolized. *Apilactobacillus ozensis* is the only known heterofermentative LAB with two types of *adhE,* encoding a 458-amino acid partially deleted AdhE and a 877-amino acid complete AdhE [5]. The complete AdhE allows D-glucose metabolism without electron acceptors. On the other hand, *A. ozensis* has similar genomic characteristics, i.e. small genome (1.48 Mbp) and small numbers of CDSs (1439 genes) and genes involved in carbohydrate transport and metabolism (62 genes), with FLAB, indicating that *A. ozensis* is genetically related to FLAB. These mean that *Apilactobacillus* spp. are generally members of FLAB, and *A. ozensis* is, however, an atypical species in the genus due to the presence of a complete *adhE* gene. *Lactobacillus kosoi*, isolated from a sugar-vegetable fermented beverage [21], was recently classified as a member of the genus *Apilactobacillus* [10] but was only recently considered a later heterotypic synonym of *A. micheneri* [22]. Recent study found a strain of *Leuconostoc citreum* showing FLAB-like poor D-glucose metabolic property, due to the absence of an active promoter region upstream of the *adhE* gene [14]. The strain showed distinct genomic characteristics from FLAB, and the strain was thus classified as pseudo-FLAB.

The reduction of genes involved in carbohydrate transport and metabolism of FLAB has been well characterized [9]. With the present study we have shown that this characteristic is generally shared amongst species of *Lactobacillaceae* with small genomes (Fig. 3). Of the 21 COG functional categories, compared on genome sizes, class G (genes involved in carbohydrate transport and metabolism) showed the largest gradient of regression (95.4), indicating that species of *Lactobacillaceae* have the ability to reduce gene numbers involved in carbohydrate transport and metabolism in accordance with reduction of genome size. The reduction rate is approx. 95 genes per 1 Mbp genomic size. All FLAB positioned below the regression line in the class G (Fig. 3b). Based on the genome sizes and a formula for class G (y = 95.452 x – 52.387), deduced gene numbers of class G in *A. micheneri*, *A. quenuiae,* and *A. timberlakei* are approx. 87, 98, and 95, respectively. Since these species only possess 54, 53, and 55 genes, respectively, this represent, in this order, only 62, 54, and 58% of the deduced gene numbers.

The observed gene reduction trend in *Lactobacillaceae* is not applicable to all classes related to metabolism. Class E (genes involved in amino acid transport and metabolism) showed a relatively large gradient value of 61.6. Other classes, i.e. class C (energy production and conversion), class F (nucleotide transport and metabolism), class H (coenzyme transport and metabolism), class I (lipid transport and metabolism), class P (inorganic ion transport and metabolism), and class Q (secondary metabolites biosynthesis, transport and catabolism), displayed low values (Fig. 3a, Supplemental Fig. S1). This would suggest that metabolism/requirement of carbohydrate and amino acids are highly dependent on species, but those of nucleotides, coenzymes, lipids, and inorganic ions and biosynthesis of secondary metabolites are not markedly changed, or not common characteristics in *Lactobacillaceae*. In fact, amino acid requirement and carbohydrate metabolic properties are diverse in the family *Lactobacillaceae*, and *Lactiplantibacillus plantarum* and *Lacticaseibacillus casei,* representatives of species possessing large genomes in the family, require small numbers of amino acids and metabolize a variety of carbohydrates [23, 24]. FLAB positioned close to the regression line in the classes C, E, F, H, I, P, and Q, suggesting that FLAB have only general gene reduction in these classes in accordance with reduction of genome sizes. The slopes of class D (genes involved in cell cycle control, cell division and chromosome partitioning), class N (cell motility), and class U (intracellular trafficking, secretion, and vesicular transport) were only 5.1, 3.8, and 3.6, respectively, indicating minor changes in gene numbers among *Lactobacillaceae* species with different genome sizes.

Since FLAB share unique metabolic properties, gene profiles used for metabolism were compared among 174 organisms in the family *Lactobacillaceae*. Surprisingly, all FLAB, i.e. *A. apinorum, A. kunkeei, A. micheneri, A. quinuiae, A. timberlakei*, and *Fructobacillus* spp., belonged to a single cluster (Fig. 4), although *Apilactobacillus* spp. and *Fructobacillus* spp. are phylogenetically distant. They were originally classified in two different families, i.e. *Lactobacillaceae* and *Leuconostocaceae* [25]*,* while these families were combined during a recent taxonomic study [10]. *Leuconostoc* spp. and *Oenococcus* spp., which are phylogenetic relatives of *Fructobacillus* spp. [9, 26], produced a single cluster, while this cluster was clearly distinct from a cluster containing *Fructobacillus* spp. The analysis of specifically missing genes in FLAB revealed that a number of genes involved in carbohydrate transport and metabolism are commonly lacked in the organisms, especially in multiple genes involved in mannose and galactose metabolic pathways. These are well correlated with their metabolic properties, meaning that they are poor carbohydrate fermenters and are not able to metabolize galactose or mannose. Bacteria remove unnecessary genes involved in metabolism at pathway level during adaptation to new environmental conditions [27]. This clearly indicates that fructose-rich environments forced the reduction of similar genes involved in metabolism to phylogenetically distant organisms, i.e. FLAB. The adaptation to new habitats, as observed for FLAB, is described as convergent evolution. Only a few studies reported a possible convergent evolution among the phylogenetically distant bacteria, which are convergent evolution between LAB and bifidobacteria by similar process of extensive loss of genes [28] and among Gram-negative pathogens for a common host infection strategy [29]. The FLAB cluster included a few non-FLAB species possessing small genomes (< 1.69 Mbp), i.e. *A. ozensis*, *Fructilactobacillus florum, Fructilactobacillus fructivorans, Fructilactobacillus lindleri,* and *Holzapfelia floricola*. These species are able to metabolize a limited number of carbohydrates [17, 30, 31]. Of the five species, *A. ozensis* and *Fructilactobacillus* spp. are phylogenetically related to the fructophilic *Apilactobacillus* spp. [17, 32], whereas one *Fructilactobacillus* species, *Fructilactobacillus sanfranciscensis*, a representative LAB for sourdough fermentation [33], positioned distantly from the cluster in the dendrogram despite their close phylogenetic relationship (Fig. 4). This might be due to different origins of the organisms, which results in different nutrition requirement. *Apilactobacillus ozensis, F. florum,* and *H. floricola* clustered with FLAB are originated from flowers [17, 30, 31], and facultative fructophilic characteristics have been reported in *F. florum* [31]. On the other hand, while several FLAB species are components of bee microbiota, other bee-associated species, i.e. species in *L. helsingborgensis* group and *B. mellis-mellifer* group [15], positioned distantly from the FLAB cluster, indicating that a single habitat has led multiple evolutions in the organisms. While FLAB lack complete PTS transporters, the bee-associated species possess over 40 complete PTS transporters [18]. Species possessing small genomes (< 1.69 Mbp) spread into several clusters, and the cluster separation is generally in agreement with their taxonomic positions. This means that even if species in the family *Lactobacillaceae* have reduced genes involved in metabolism in accordance with reduction of genome size, gene contents are generally conserved in each phylogenetic group. However, this is not applicable to *Fructobacillus* spp., as described above.

Two genes related to bacterial stress tolerance were also commonly ‘missing’ in FLAB (Table 5). One of the genes, *htpX*, has been involved in resistance to heavy metal, cadmium, in *Escherichia coli* [34], and *zntA* encoding Cd^2+^/Zn^2+^-exporting ATPase, which also confers cadmium tolerance in *E. coli* [35], was also a ‘missing’ gene in FLAB, suggesting that FLAB are possibly sensitive to cadmium. Interestingly, *A. kunkeei* reduced population in gut microbiota of honey bees exposed to cadmium [36]. Fructophilic *Apilactobacillus* spp.*,* i.e. *A. apinorum, A. kunkeei, A. micheneri, A. quenuiae,* and *A. timberlakei,* removed less amounts of cadmium than the other honey bee commensal *Lactobacillaceae* species in a broth supplemented with cadmium [36]. Cadmium is one of the industrial pollutants, which has been found in croplands [37], one of the working areas of honey bees. Based on the habitats, FLAB originally had few chances to contact cadmium, but FLAB might have a risk of exposure to the heavy metal in gut of honey bees at present. Adaptation to this environmental change of the habitats might influence evolution of FLAB in a future.

## Conclusion

Genomic and phenotypic characteristics revealed that *A, micheneri, A. quenuiae,* and *A. timberlakei* had undergone a similar reductive evolution with known FLAB in order to adapt to fructose-rich environments and are novel members of FLAB. *Lactobacillaceae* organisms have reduced genes involved in carbohydrate transport and metabolism in accordance with reduction of genome size and FLAB have been characterized to reduce more genes involved in carbohydrate transport and metabolism than other species in *Lactobacillaceae*. Pan-genome analysis of genes involved in metabolism suggested that fructose-rich environments have induced similar evolution in phylogenetically distant FLAB species, and this would be an example of convergent evolution of LAB.

## Material and methods

### Bacterial strains and culture conditions

*Apilactobacillus micheneri* JCM 33323^T^, *A quenuiae* JCM 33324^T^, and *A. timberlakei* JCM 33325^T^ were obtained from Japan Collection of Microorganisms (JCM). These strains were cultured in D-fructose (1%, w/v)-yeast-peptone (FYP) broth at 30 °C for 24 h, as described previously [1].

### Biochemical characterization

Growth characteristics on D-fructose and D-glucose and the requirement of external electron acceptors for D-glucose dissimilation were determined in FYP broth, GYP broth, and GYP broth supplemented with 1% (w/v) sodium pyruvate (GYP-P), as previously described [1]. GYP broth differed from FYP broth by containing 1% (w/v) D-glucose instead of D-fructose [1]. Accumulation of lactate, acetate, and ethanol from D-glucose metabolism was determined after 2 days of incubation at 30 °C in GYP broth by high performance liquid chromatography (HPLC), as described previously [38]. Mannitol production from D-fructose was determined in FGYP broth using a high performance anion exchange chromatography coupled with pulsed amperometric detection (HPAEC-PAD) system (model ICS-3000, Dionex, United Kingdom), as described previously [38]. FGYP broth contains 1% (w/v) each of D-fructose and D-glucose. Carbohydrate fermentation reactions were recorded using API CHL galleries (bioMérieux, Marcyl’Etoile, France), according to the manufacturer’s instructions. Readings were taken for 7 days at 30 °C.

ADH, ALDH, and NADH oxidase activities were determined in cells of *A. micheneri, A. quenuiae,* and *A. timberlakei*. Culturing of the cells, preparation of cell-free extracts, and enzyme assays were performed as described by Maeno et al. [5].

### Genomic data of *A. micheneri*, *A. quenuiae, A. timberlakei*, and related species

The draft genome sequences of *A. micheneri*, *A. quenuiae* and *A. timberlakei* were obtained from the RefSeq database at NCBI or the DFAST Archive of Genome Annotation (DAGA, https://dfast.nig.ac.jp) [39]. The annotated draft genome sequences or complete genome sequences of 171 strains of FLAB and other LAB were also obtained from the RefSeq or DAGA, which consist of 164 species and 11 subspecies in 20 genera of the family *Lactobacillaceae*. The species were selected based on a recent taxonomic study of the genus *Lactobacillus* and related taxa by Salvetti et al. [32] which separated the genus *Lactobacillus* into ten phylogenetic groups, and representative species in the ten phylogenetic groups were included. *Leuconostoc* spp. and *Oenococcus* spp. were also included because of their close phylogenetic relationships with *Fructobacillus* spp. Strains included are summarized in Supplemental Table S1. All genome data included in the analysis were annotated using the DFAST pipeline.

### Phylogenetic analysis

Orthologous clusters that were conserved in the 174 strains of FLAB and other species in the family *Lactobacillaceae* and *Lactococcus lactis* IL1403 (NC_002662.1, included as the out-group) were determined by GET_HOMOLOGUES software (version 1.3) based on the all-against-all bidirectional BLAST alignment and the MCL graph-based algorithm [40]. The amino acid sequences within each cluster were aligned using MUSCLE (version 3.8.31) [41]. Poorly aligned or divergent regions were trimmed using Gblocks [42], and conserved regions were then concatenated using FASconCAT-G [43]. A partitioned maximum likelihood analysis was performed to construct the phylogenetic tree with RAxML (version 8.1.22) [44] using the bestfit evolutionary models predicted for each alignment by ProtTest [45]. The number of bootstrapping was 1000 replicates.

### Genome analysis

For functional comparison of the gene contents among the 174 strains, CDSs predicted in each strain were assigned to COG functional classification using the COGNITOR software [46]. For revealing correlation between genome size (Mbp) and number of genes in each COG class, gradients of the regression lines were obtained using Microsoft Excel 365. The metabolic pathway for each strain was also predicted using the KEGG Automatic Annotation Service (KAAS) by assigning KEGG Orthology (KO) numbers to each predicted CDS [47]. Numbers of CDSs assigned with each KO number were counted using an in-house Python script and subjected to a hierarchical clustering analysis using the hclust package in R (http://www.r-project.org). A dendrogram was produced based on presence/absence of genes involved in metabolism of KAAS. To study evolutional trend of *Lactobacillaceae* species, species possessing small genomes (< 1.69 Mbp, the largest genome of FLAB, *Fructobacillus tropaeoli*) were highlighted in the analyses, which contained 32 species in 11 genera (Table 2).

For further characterization of metabolic systems in FLAB (*n* = 10), ‘specific’ and ‘missing’ genes involved in metabolism were identified. Genes extracted by the KAAS prediction as described above were included in this study. FLAB ‘specific’ genes were defined as conserved genes in greater than 80% of FLAB species (conserved in nine or ten strains) but in less than 20% of the other species in the *Lactobacillaceae* included (*n* = 164, conserved in 32 strains or less), and ‘missing’ genes were defined as genes conserved in less than 20% of FLAB species (conserved in one or zero strains) but conserved in greater than 80% of the other species in the *Lactobacillaceae* included (conserved in 132 strains or more). For finding ‘specific’ and ‘missing’ genes, we consider a simple probabilistic model. Suppose that a gene is randomly lost with probability *x* in each single species from a common ancestor with all genes. Then the probabilities of being lost in > 8 species (out of 10), and being retained by > 132 species (out of 164) are ${\sum}_{k=0}^1\left(\genfrac{}{}{0pt}{}{10}{k}\right){\left(1-x\right)}^k{x}^{10-k}$ and ${\sum}_{k=0}^{32}\left(\genfrac{}{}{0pt}{}{164}{k}\right){x}^k{\left(1-x\right)}^{164-k}$, respectively. When these values are multiplied, the resulting function depicts a single sharp peak of height 5e-0.5 around *x* = 0.2. This implies that several genes may be chosen by chance when we consider ~ 10^5^ genes. Since the number of pan-genome genes involved in metabolism of the 174 strains determined in KAAS was 2340, genes were selected not by chance especially when they are involved in related metabolic pathways.

## Supplementary Information

**Additional file 1: Supplemental Table S1.** List of strains in genomic analysis.

**Additional file 2: Supplemental Figure S1.** Correlation between genome sizes and number of genes assigned in each COG class. One-hundred and seventy-four strains of Lactobacillaceae were included in the study. FLAB were marked with red points.

## Data Availability

All genomic data used in the present study are available at the public database.

## Abbreviations

FLAB: Fructophilic lactic acid bacteria
LAB: Lactic acid bacteria
ADH: Alcohol dehydrogenase
ALDH: Acetaldehyde dehydrogenase
CDS: Coding DNA sequences
COG: Cluster of orthologous groups
KAAS: KEGG Automatic Annotation Server
PTS: Phosphotransferase system
