# Supplementary material for: Unique niche-specific adaptation of fructophilic lactic acid bacteria and proposal of three Apilactobacillus species as novel members of the group
Source: BMC Microbiol. 2021 Feb 9;21:41. doi: 10.1186/s12866-021-02101-9 (PMC7871557; doi:10.1186/s12866-021-02101-9)
Supplement: Supplementary file 1 — Additional file 1: Supplemental Table S1. List of strains in genomic analysis. [file 12866_2021_2101_MOESM1_ESM.pdf]

Supplemental Table S1. List of strains in genomic analysis

DAGA ID/Accession No.	Genus	Species	Subspecies	Strain	Genome size (Mbp)	GC%	No. of CDSs
**SRR1151200**	*Agrilactobacillus*	*composti*		DSM 18527	3.46	44.0	3,306
**GCA_001436185.1**	*Amylolactobacillus*	*amylophilus*		DSM 20533	1.56	43.6	1,561
**ERR387486**	*Amylolactobacillus*	*amylotrophicus*		DSM 20534	1.60	42.6	1,602
**GCA_001281175.1**	*Apilactobacillus*	*apinorum*		Fhon13	1.46	34.6	1,315
**GCA_001281265.1**	*Apilactobacillus*	*kunkeei*		YH-15	1.52	36.4	1,353
**NZ_POSO00000000**	*Apilactobacillus*	*micheneri*		Hlig3	1.46	30.7	1,485
**ERR433479**	*Apilactobacillus*	*ozensis*		DSM 23829	1.48	31.9	1,439
**NZ_POSN00000000**	*Apilactobacillus*	*quenuiae*		HV 6	1.58	30.3	1,553
**NZ_POST00000000**	*Apilactobacillus*	timberlakei		HV 12	1.54	30.5	1,570
**GCA_000970795.1**	*Bombilactobacillus*	*mellifer*		Bin4	1.82	39.3	1,661
**GCA_000967245.1**	*Bombilactobacillus*	*mellis*		Hon2	1.81	36.2	1,650
**ERR387471**	*Companilactobacillus*	*alimentarius*		DSM 20249	2.33	35.4	2,232
**ERR387491**	*Companilactobacillus*	*crustorum*		LMG 23699	2.22	35.0	2,165
**GCA_001434775.1**	*Companilactobacillus*	*farciminis*		DSM 20184	2.48	36.4	2,417
**ERR387495**	*Companilactobacillus*	*futsaii*		JCM 17355	2.49	35.6	2,449
**GCA_001050475.1**	*Companilactobacillus*	*ginsenosidimutans*		EMML 3141	2.59	36.7	2,558
**GCA_000831645.3**	*Companilactobacillus*	*heilongjiangensis*		DSM 28069	2.79	37.5	2,485
**ERR387524**	*Companilactobacillus*	*mindensis*		DSM 14500	2.33	38.2	2,205
**ERR433477**	*Companilactobacillus*	*nantensis*		DSM 16982	2.92	36.2	2,774
**ERR387514**	*Companilactobacillus*	*nodensis*		DSM 19682	2.68	37.6	2,651
**ERR387521**	*Companilactobacillus*	*paralimentarius*		DSM 13238	2.53	35.1	2,445
**GCA_001434665.1**	*Companilactobacillus*	*tucceti*		DSM 20183	2.17	34.1	2,103
**GCA_001434295.1**	*Companilactobacillus*	*versmoldensis*		DSM 14857	2.37	38.3	2,330
**GCA_001434695.1**	*Dellaglioa*	*algida*		DSM 15638	1.59	36.0	1,531
**GCA_001436645.1**	*Fructilactobacillus*	*florum*		DSM 22689	1.35	41.1	1,302
**SRR1151190**	*Fructilactobacillus*	*fructivorans*		DSM 20203	1.37	38.9	1,336
**GCA_001311115.1**	*Fructilactobacillus*	*lindneri*		JCM 11027	1.44	34.1	1,632
**GCA_000225325.1**	*Fructilactobacillus*	*sanfranciscensis*		TMW 1.1304	1.38	34.8	1,402

**NZ_FOLI00000000**	*Fructobacillus*	durionis		DSM 19113	1.33	44.7	1,255
**NZ_BBXQ00000000**	*Fructobacillus*	ficulneus		JCM 12225	1.54	43.9	1,427
**NZ_BBXR00000000**	*Fructobacillus*	fructosus		NRIC 1058	1.49	44.5	1,465
**NZ_FNWS00000000**	*Fructobacillus*	pseudoficulneus		DSM 15468	1.41	44.5	1,323
**NZ_BBXT00000000**	*Fructobacillus*	tropaeoli		F214-1	1.69	44.2	1,625
**GCA_000428925.1**	*Furfurilactobcillus*	*rossiae*		DSM 15814	2.86	43.3	2,700
**ERR387541**	*Furfurilactobcillus*	*siliginis*		DSM 22696	2.04	44.1	1,980
**GCA_001436605.1**	*Holzapfelia*	*floricola*		DSM 23037	1.29	34.5	1,252
**GCA_001436115.1**	*Lacticaseibacillus*	*brantae*		DSM 23927	1.93	47.5	1,900
**SRR1151192**	*Lacticaseibacillus*	*camelliae*		DSM 22697	2.56	55.4	2,402
**GCA_000829055.1**	*Lacticaseibacillus*	*casei*		ATCC 393	2.95	47.9	2,890
**ERR387460**	*Lacticaseibacillus*	*manihotivorans*		DSM 13343	3.08	47.7	3,012
**ERR387517**	*Lacticaseibacillus*	*nasuensis*		JCM 17158	2.27	57.0	2,137
**GCA_001311175.1**	*Lacticaseibacillus*	*pantheris*		JCM 12539	2.53	52.8	3,068
**ERR433482**	*Lacticaseibacillus*	*paracasei*	*tolerans*	LMG 9191	2.45	46.3	2,455
**GCA_000829035.1**	*Lacticaseibacillus*	*paracasei*	*paracasei*	JCM 8130	3.02	46.6	2,945
**GCA_000026505.1**	*Lacticaseibacillus*	*rhamnosus*		GG (ATCC 53103)	3.01	46.7	2,817
**GCA_001311785.1**	*Lacticaseibacillus*	*saniviri*		JCM 17471	2.42	47.8	3,308
**ERR387540**	*Lacticaseibacillus*	*sharpeae*		DSM 20505	2.44	53.4	2,344
**GCA_001436135.1**	*Lacticaseibacillus*	*thailandensis*		DSM 22698	2.06	53.5	1,893
**GCA_001433745.1**	*Lacticaseibacillus*	*zeae*		DSM 20178	3.12	47.7	2,961
**ERR387476**	*Lactiplantibacillus*	*fabifermentans*		DSM 21115	3.27	45.0	3,111
**GCA_001039045.1**	*Lactiplantibacillus*	*herbarum*		TCF032-E4	2.90	43.5	2,805
**GCA_000271445.1**	*Lactiplantibacillus*	*pentosus*		KCA1	3.43	46.4	3,039
**SRR1151228**	*Lactiplantibacillus*	*plantarum*	*argentoratensis*	DSM 16365	3.17	45.0	2,936
**GCA_000203855.3**	*Lactiplantibacillus*	*plantarum*	*plantarum*	WCFS1	3.35	44.4	3,123
**GCA_001438845.1**	*Lactiplantibacillus*	*xiangfangensis*		LMG 26013	2.99	45.1	2,757
**ERR387527**	*Lactobacillus*	*acetotolerans*		DSM 20749	1.57	36.2	1,518
**GCA_000011985.1**	*Lactobacillus*	*acidophilus*		NCFM	1.99	34.7	1,875
**GCA_000178475.1**	*Lactobacillus*	*amylolyticus*		DSM 11664	1.54	38.3	1,567

**GCA_000970735.1**	*Lactobacillus*	*apis*		Hma11	1.72	36.6	1,564
**GCA_001434005.1**	*Lactobacillus*	*crispatus*		DSM 20584	2.06	36.6	2,017
**ERR433469**	*Lactobacillus*	*delbrueckii*	*lactis*	LMG 7942	1.86	49.7	1,802
**ERR433470**	*Lactobacillus*	*delbrueckii*	*indicus*	DSM 15996	1.85	49.6	1,804
**ERR433468**	*Lactobacillus*	*delbrueckii*	*delbrueckii*	DSM 20074	1.72	50.1	1,702
**GCA_000014405.1**	*Lactobacillus*	*delbrueckii*	*bulgaricus*	ATCC BAA-365	1.86	49.7	1,906
**GCA_000387565.1**	*Lactobacillus*	*delbrueckii*	*jakobsenii*	ZN7a-9	1.73	50.2	1,677
**GCA_000312645.1**	*Lactobacillus*	*equicursoris*		CIP 110162	2.15	47.8	1,996
**ERR387508**	*Lactobacillus*	*gallinarum*		DSM 10532	1.93	36.5	1,912
**GCA_000014425.1**	*Lactobacillus*	*gasseri*		ATCC 33323	1.89	35.3	1,808
**GCA_000296855.1**	*Lactobacillus*	*gigeriorum*		CRBIP 24.85	1.93	36.9	1,891
**ERR387507**	*Lactobacillus*	*hamsteri*		DSM 5661	1.79	35.1	1,712
**GCA_000761135.1**	*Lactobacillus*	*helsingborgensis*		wkB8	1.93	36.7	1,735
**GCA_000422165.1**	*Lactobacillus*	*helveticus*		CNRZ32	2.23	36.9	2,407
**GCA_000160875.1**	*Lactobacillus*	*iners*		DSM 13335	1.28	32.5	1,191
**ERR387510**	*Lactobacillus*	*intestinalis*		DSM 6629	1.99	35.3	1,838
**SRR1151162**	*Lactobacillus*	*jensenii*		DSM 20557	1.62	34.3	1,478
**GCA_000008065.1**	*Lactobacillus*	*johnsonii*		NCC 533	1.99	34.6	1,875
**ERR387511**	*Lactobacillus*	*kalixensis*		DSM 16043	2.07	36.1	1,937
**ERR387484**	*Lactobacillus*	*kefiranofaciens*	*kefirgranum*	DSM 10550	2.03	37.4	2,020
**GCA_000214785.1**	*Lactobacillus*	*kefiranofaciens*	*kefiranofaciens*	ZW3	2.35	37.4	2,420
**GCA_000970755.1**	*Lactobacillus*	*kimbladii*		Hma2	2.19	35.8	1,972
**GCA_000615285.1**	*Lactobacillus*	*kitasatonis*		JCM 1039	1.90	37.5	2,048
**GCA_000967195.1**	*Lactobacillus*	*kullabergensis*		Biut2	2.12	35.5	1,943
**GCA_000970775.1**	*Lactobacillus*	*melliventris*		Hma8	2.12	35.8	1,994
**ERR433480**	*Lactobacillus*	*panis*		DSM 6035	1.99	48.1	1,888
**GCA_000297025.1**	*Lactobacillus*	*pasteurii*		CRBIP 24.76	1.91	38.6	1,807
**GCA_000425905.1**	*Lactobacillus*	*psittaci*		DSM 15354	1.54	35.7	1,340
**ERR387546**	*Lactobacillus*	*taiwanensis*		DSM 21401	1.87	33.9	1,816
**GCA_000159415.1**	*Lactobacillus*	*ultunensis*		DSM 16047	2.25	36.0	2,117

**ERR433467**	*Lapidilactobacillus*	*concavus*		DSM 17758	1.88	43.3	1,747
**SRR1151201**	*Lapidilactobacillus*	*dextrinicus*		DSM 20335	1.81	38.0	1,725
**SRR1151125**	*Latilactobacillus*	*curvatus*		DSM 20019	1.81	42.0	1,814
**ERR387479**	*Latilactobacillus*	*fuchuensis*		DSM 14340	2.11	41.8	2,016
**ERR387528**	*Latilactobacillus*	*graminis*		DSM 20719	1.83	40.3	1,739
**GCA_000026065.1**	*Latilactobacillus*	*sakei*	*sakei*	23K	1.88	41.3	1,849
**GCA_000298115.2**	*Lentilactobacillus*	*buchneri*		CD034	2.56	44.2	2,424
**GCA_001434255.1**	*Lentilactobacillus*	*diolivorans*		DSM 14421	3.26	40.0	3,011
**ERR387467**	*Lentilactobacillus*	*farraginis*		DSM 18382	2.79	42.0	2,670
**GCA_000159315.1**	*Lentilactobacillus*	*hilgardii*		ATCC 8290	2.72	39.6	2,548
**ERR387463**	*Lentilactobacillus*	*kefiri*		DSM 20587	2.32	41.7	2,208
**GCA_001434135.1**	*Lentilactobacillus*	*kisonensis*		DSM 19906	3.01	41.7	2,758
**GCA_001311395.1**	*Lentilactobacillus*	*otakiensis*		JCM 15040	2.34	42.3	3,025
**GCA_001435315.1**	*Lentilactobacillus*	*parabuchneri*		DSM 5707	2.57	43.4	2,373
**ERR387516**	*Lentilactobacillus*	*rapi*		DSM 19907	2.85	42.9	2,643
**GCA_001436555.1**	*Lentilactobacillus*	*senioris*		DSM 24302	1.57	39.1	1,568
**ERR387545**	*Lentilactobacillus*	*sunkii*		DSM 19904	2.71	42.1	2,561
**NC_018673-75,698-699**	*Leuconostoc*	carnosum		JB16	1.77	37.1	1,699
**NC_010466,67-71**	*Leuconostoc*	citreum		KM20	1.77	38.9	1,856
**NZ_AEIZ00000000**	*Leuconostoc*	fallax		KCTC 357	1.64	37.5	1,890
**NZ_AEMI00000000**	*Leuconostoc*	gelidum		KCTC3527	1.96	36.6	1,900
**NC_014131-36**	*Leuconostoc*	kimchii		MSNU 1154	2.1	37.9	2,087
**NZ_CP016598-601**	*Leuconostoc*	lactis		Wikin40	1.79	43.1	1,695
**NC_008531,496**	*Leuconostoc*	mesenteroides		ATCC 8293	2.04	37.7	1,981
**NZ_JAUI00000000**	*Leuconostoc*	pseudomesenteroides		1159	2.04	39.0	2,075
**ERR387483**	*Levilactobacillus*	*acidifarinae*		DSM 19394	2.91	51.6	2,738
**GCA_000014465.1**	*Levilactobacillus*	*brevis*		ATCC 367	2.34	46.1	2,281
**ERR387482**	*Levilactobacillus*	*hammesii*		DSM 16381	2.81	49.4	2,591
**ERR433476**	*Levilactobacillus*	*namurensis*		DSM 19117	2.47	52.0	2,227
**GCA_001434055.1**	*Levilactobacillus*	*parabrevis*		ATCC 53295	2.61	49.0	2,363

**GCA_001437125.1**	*Levilactobacillus*	*paucivorans*		DSM 22467	2.36	49.1	2,210
**GCA_001436675.1**	*Levilactobacillus*	*senmaizukei*		DSM 21775	2.22	48.6	2,122
**ERR387543**	*Levilactobacillus*	*spicheri*		DSM 15429	2.74	55.9	2,451
**GCA_001434115.1**	*Levilactobacillus*	*zymae*		DSM 19395	2.70	53.6	2,456
**ERR387498**	*Ligilactobacillus*	*agilis*		DSM 20509	2.05	41.7	2,015
**ERR387553**	*Ligilactobacillus*	*animalis*		DSM 20602	1.87	41.1	1,812
**ERR433462**	*Ligilactobacillus*	*apodemi*		DSM 16634	2.08	38.6	2,019
**GCA_001435375.1**	*Ligilactobacillus*	*araffinosus*		DSM 20653	1.48	38.1	1,414
**ERR387530**	*Ligilactobacillus*	*aviarius*		DSM 20655	1.67	40.1	1,585
**ERR387509**	*Ligilactobacillus*	*equi*		DSM 15833	2.24	39.0	2,159
**ERR387461**	*Ligilactobacillus*	*hayakitensis*		DSM 18933	1.64	34.0	1,543
**ERR387504**	*Ligilactobacillus*	*murinus*		DSM 20452	2.16	40.0	2,030
**GCA_000349725.1**	*Ligilactobacillus*	*pobuzihii*		E100301	2.35	37.7	2,127
**GCA_000224985.1**	*Ligilactobacillus*	*ruminis*		ATCC 27782	2.07	43.5	1,978
**GCA_000008925.1**	*Ligilactobacillus*	*salivarius*		UCC118	2.13	33.0	2,101
**GCA_000160835.1**	*Limosilactobacillus*	*antri*		DSM 16041	2.30	51.1	2,128
**SRR1151138**	*Limosilactobacillus*	*equigenerosi*		DSM 18793	1.60	42.7	1,545
**GCA_000397165.1**	*Limosilactobacillus*	*fermentum*		F-6	2.06	51.7	2,022
**GCA_001436045.1**	*Limosilactobacillus*	*frumenti*		DSM 13145	1.73	42.6	1,679
**GCA_001434365.1**	*Limosilactobacillus*	*gastricus*		DSM 16045	1.85	41.6	1,819
**GCA_001293735.1**	*Limosilactobacillus*	*gorillae*		KZ01	1.64	48.1	1,568
**ERR387499**	*Limosilactobacillus*	*ingluviei*		DSM 15946	2.14	49.9	2,086
**GCA_001436025.1**	*Limosilactobacillus*	*mucosae*		DSM 13345	2.25	46.4	2,014
**GCA_001434465.1**	*Limosilactobacillus*	*oris*		DSM 4864	2.03	50.0	1,925
**SRR1151252**	*Limosilactobacillus*	*pontis*		DSM 8475	1.66	53.5	1,614
**GCA_000010005.1**	*Limosilactobacillus*	*reuteri*		JCM 1112	2.04	38.9	2,020
**GCA_001437055.1**	*Limosilactobacillus*	*secaliphilus*		DSM 17896	1.65	47.7	1,503
**SRR1151175**	*Limosilactobacillus*	*vaginalis*		DSM 5837	1.78	40.5	1,733
**GCA_001436755.1**	*Liquorilactobacillus*	*aquaticus*		DSM 21051	2.41	37.4	2,213
**GCA_001436735.1**	*Liquorilactobacillus*	*cacaonum*		DSM 21116	1.92	33.9	1,824

**GCA_001434915.1**	*Liquorilactobacillus*	*capillatus*		DSM 19910	2.23	37.6	2,102
**ERR387502**	*Liquorilactobacillus*	*ghanensis*		DSM 18630	2.60	37.1	2,417
**ERR387525**	*Liquorilactobacillus*	*hordei*		DSM 19519	2.29	34.8	2,239
**ERR387505**	*Liquorilactobacillus*	*nagelii*		DSM 13675	2.49	36.7	2,409
**ERR433495**	*Liquorilactobacillus*	*satsumensis*		DSM 16230	2.63	39.9	2,441
**ERR485115**	*Liquorilactobacillus*	*sucicola*		DSM 21376	2.46	38.5	2,265
**ERR387550**	*Liquorilactobacillus*	*uvarum*		DSM 19971	2.67	36.9	2,525
**GCA_001435395.1**	*Liquorilactobacillus*	*vini*		DSM 20605	2.24	37.5	2,111
**GCA_001433765.1**	*Loigolactobacillus*	*coryniformis*	*coryniformis*	DSM 20001	2.71	42.9	2,579
**ERR433491**	*Loigolactobacillus*	*rennini*		DSM 20253	2.26	40.7	2,219
**NZ_CM001398-9**	*Oenococcus*	kitaharae		DSM 17330	1.84	42.7	1,835
**NC_008528**	*Oenococcus*	oeni		PSU-1	1.78	37.9	1,836
**SRR1562044**	*Paralactobacillus*	*selangorensis*		DSM 13344	2.08	46.4	2,065
**GCA_000829395.1**	*Paucilactobacillus*	*hokkaidonensis*		LOOC260	2.40	38.2	2,328
**ERR387501**	*Paucilactobacillus*	*vaccinostercus*		DSM 20634	2.55	43.5	2,440
**GCA_000876205.1**	*Paucilactobacillus*	*wasatchensis*		WDC04	1.90	39.8	1,807
**GCA_000425885.1**	*Schleiferilactobacillus*	*harbinensis*		DSM 16991	3.13	53.1	3,049
**ERR387519**	*Schleiferilactobacillus*	*perolens*		DSM 12744	3.27	49.2	3,103
**GCA_000469325.1**	*Schleiferilactobacillus*	*shenzhenensis*		LY-73	3.27	56.4	2,975
**GCA_001435975.1**	*Secundilactobacillus*	*collinoides*		DSM 20515	3.62	46.1	3,224
**GCA_001433995.1**	*Secundilactobacillus*	*kimchicus*		JCM 15530	2.59	46.6	2,522
**SRR1151262**	*Secundilactobacillus*	*malefermentans*		DSM 5705	2.05	41.0	2,013
**ERR433478**	*Secundilactobacillus*	*odoratitofui*		DSM 19909	2.75	44.2	2,403
**GCA_001311525.1**	*Secundilactobacillus*	*oryzae*		JCM 18671	1.84	42.8	2,559
**ERR433483**	*Secundilactobacillus*	*paracollinoides*		DSM 15502	3.40	46.9	3,146
**ERR387542**	*Secundilactobacillus*	*similis*		DSM 23365	3.45	47.0	3,084
