# Supplementary material for: Unique niche-specific adaptation of fructophilic lactic acid bacteria and proposal of three Apilactobacillus species as novel members of the group
Source: BMC Microbiol. 2021 Feb 9;21:41. doi: 10.1186/s12866-021-02101-9 (PMC7871557; doi:10.1186/s12866-021-02101-9)
Supplement: Supplementary file 2 — Additional file 2: Supplemental Figure S1. Correlation between genome sizes and number of genes assigned in each COG class. One-hundred and seventy-four strains of Lactobacillaceae were included in the study. FLAB were marked with red points. [file 12866_2021_2101_MOESM2_ESM.pptx]

## Slide 1
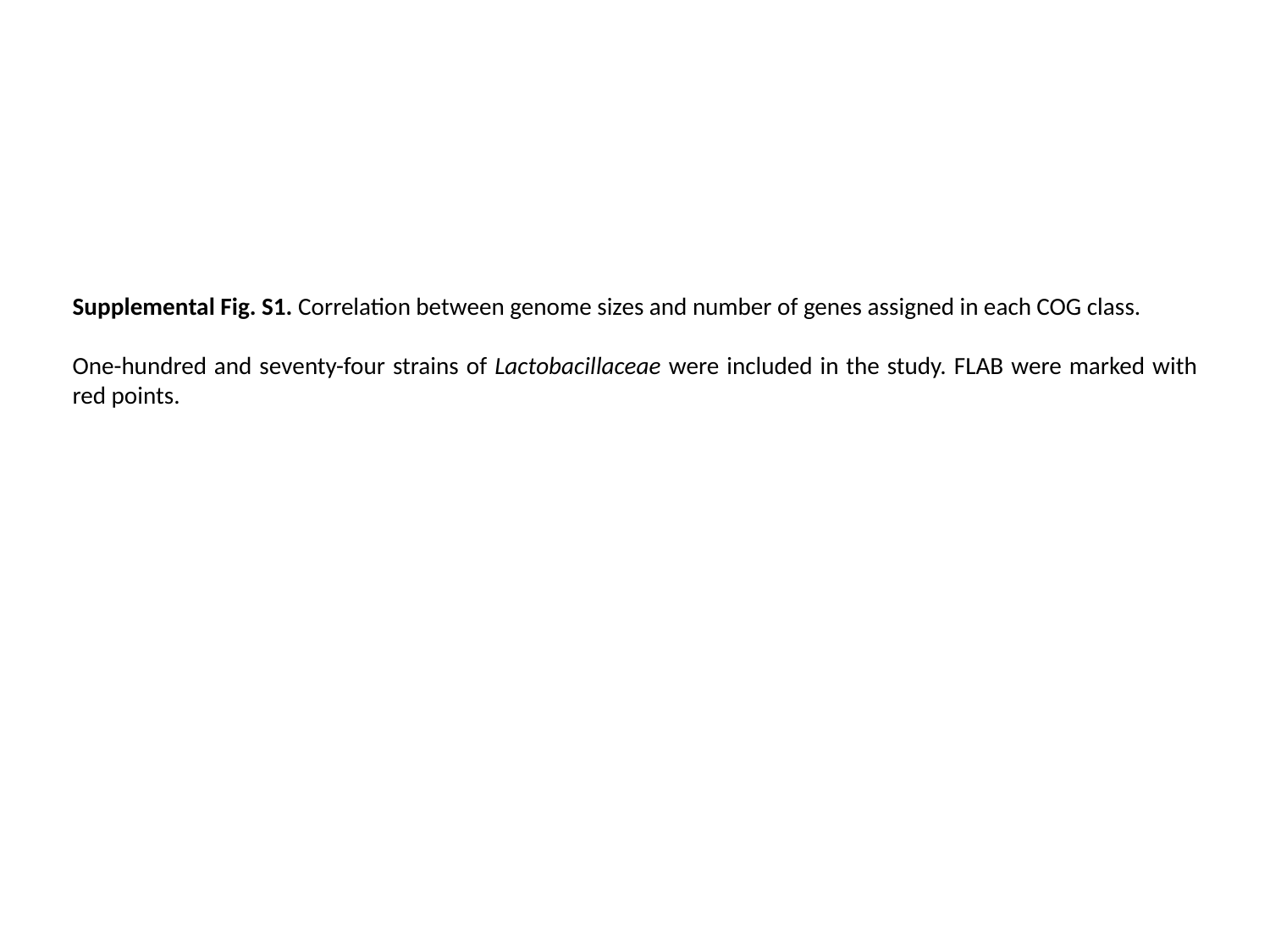

Supplemental Fig. S1. Correlation between genome sizes and number of genes assigned in each COG class.
One-hundred and seventy-four strains of Lactobacillaceae were included in the study. FLAB were marked with red points.

## Slide 2
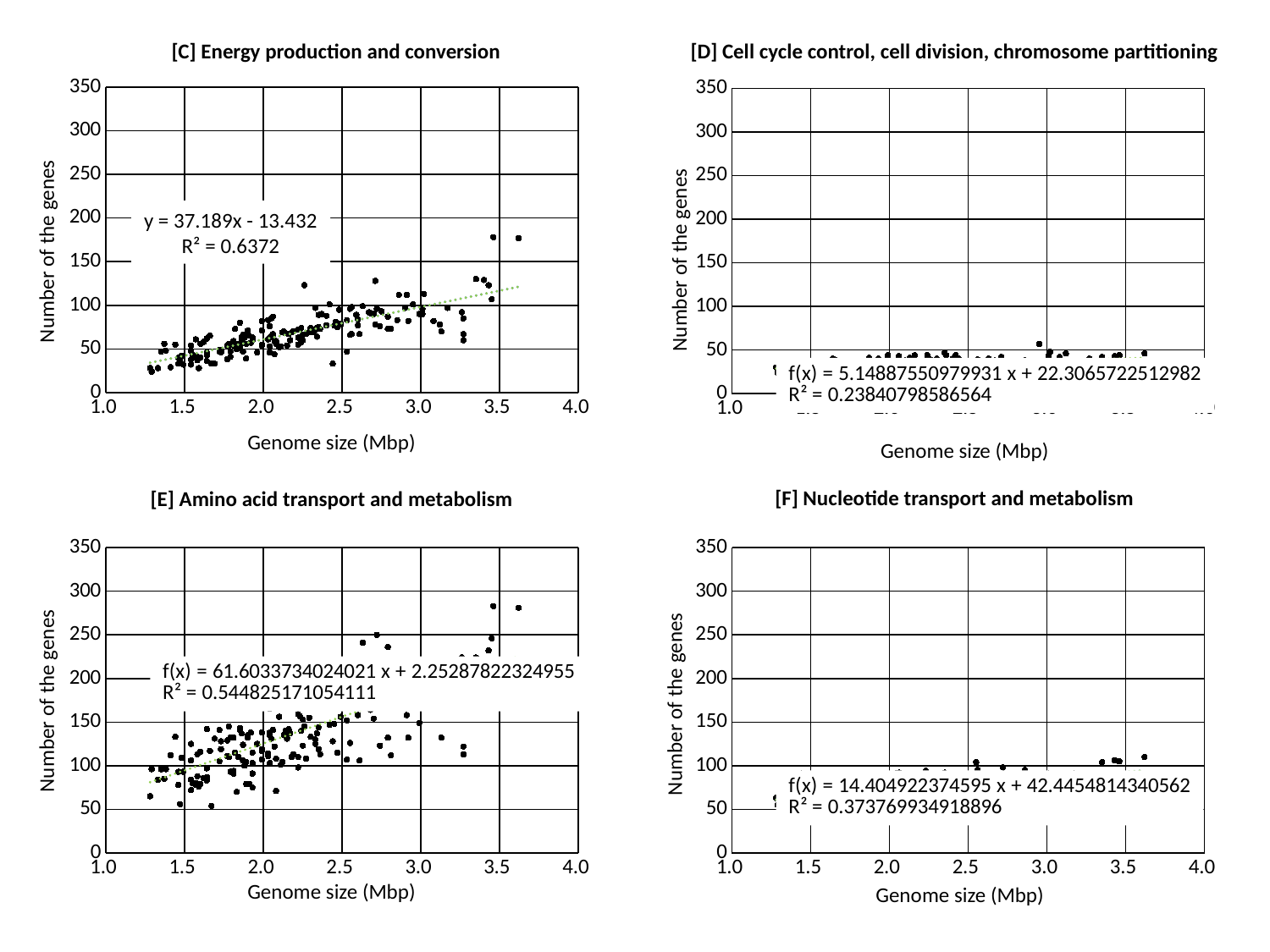

[C] Energy production and conversion
[D] Cell cycle control, cell division, chromosome partitioning
### Chart
| Category | [C]_Energy_production_and_conversion |
|---|---|
### Chart
| Category | [D]_Cell_cycle_control,_cell_division,_chromosome_partitioning |
|---|---|y = 37.189x - 13.432
R² = 0.6372
Number of the genes
Number of the genes
Genome size (Mbp)
Genome size (Mbp)
[F] Nucleotide transport and metabolism
[E] Amino acid transport and metabolism
### Chart
| Category | [E]_Amino_acid_transport_and_metabolism |
|---|---|
### Chart
| Category | [F]_Nucleotide_transport_and_metabolism |
|---|---|Number of the genes
Number of the genes
Genome size (Mbp)
Genome size (Mbp)

## Slide 3
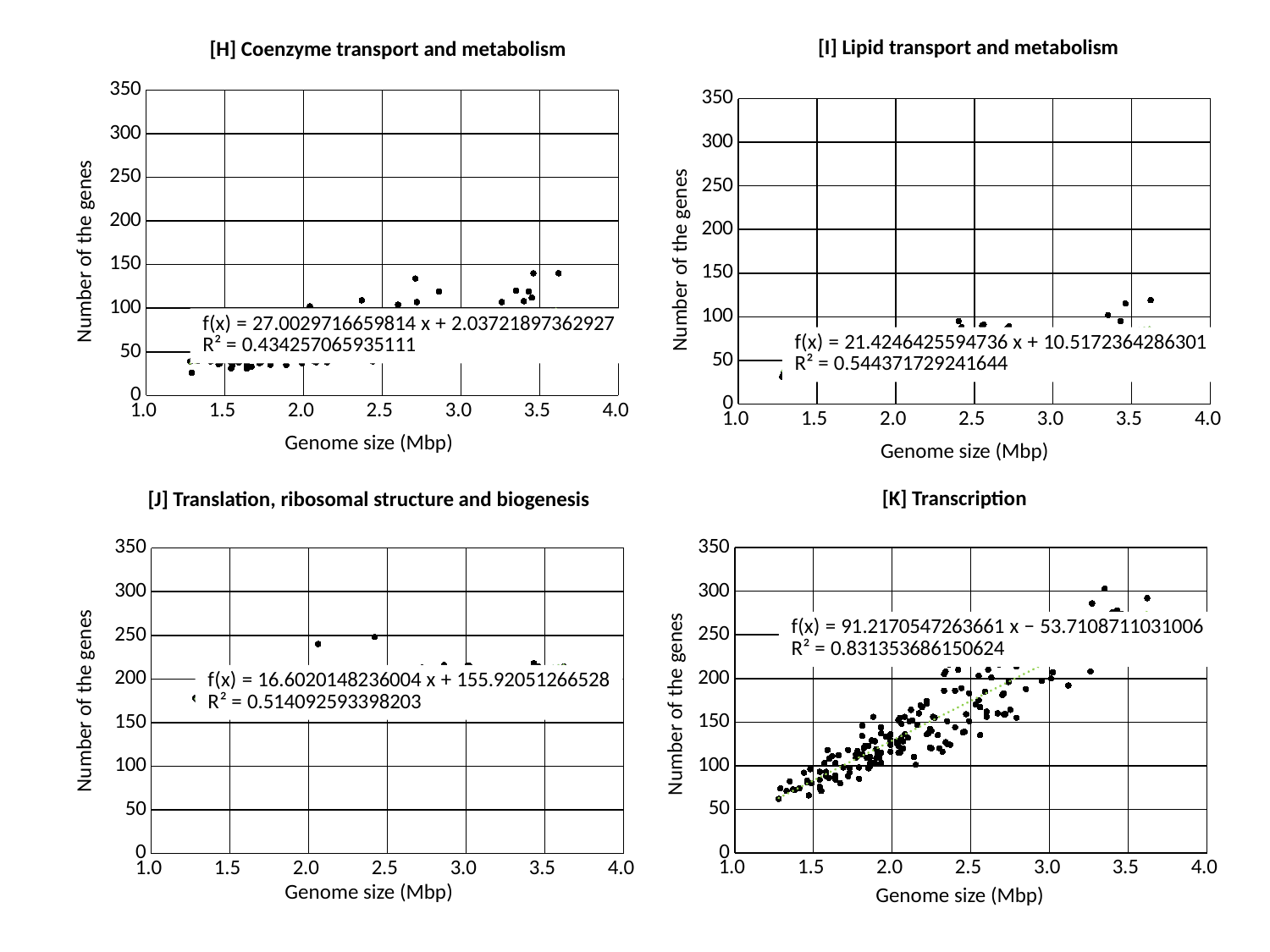

[I] Lipid transport and metabolism
[H] Coenzyme transport and metabolism
### Chart
| Category | [H]_Coenzyme_transport_and_metabolism |
|---|---|
### Chart
| Category | [I]_Lipid_transport_and_metabolism |
|---|---|Number of the genes
Number of the genes
Genome size (Mbp)
Genome size (Mbp)
[K] Transcription
[J] Translation, ribosomal structure and biogenesis
### Chart
| Category | [K]_Transcription |
|---|---|
### Chart
| Category | [J]_Translation,_ribosomal_structure_and_biogenesis_ |
|---|---|Number of the genes
Number of the genes
Genome size (Mbp)
Genome size (Mbp)

## Slide 4
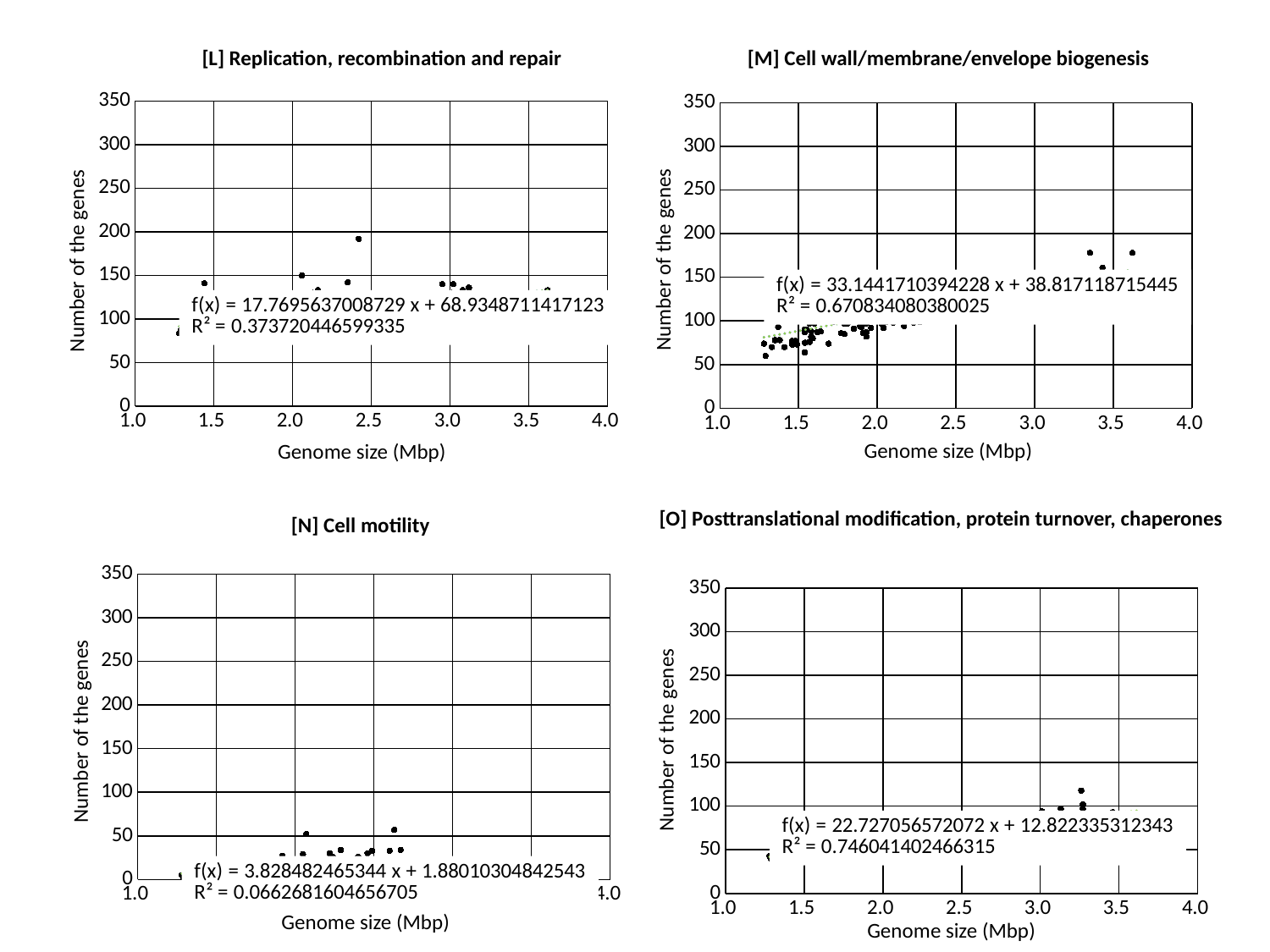

[L] Replication, recombination and repair
[M] Cell wall/membrane/envelope biogenesis
### Chart
| Category | [L]_Replication,_recombination_and_repair |
|---|---|
### Chart
| Category | [M]_Cell_wall/membrane/envelope_biogenesis |
|---|---|Number of the genes
Number of the genes
Genome size (Mbp)
Genome size (Mbp)
[O] Posttranslational modification, protein turnover, chaperones
[N] Cell motility
### Chart
| Category | [N]_Cell_motility |
|---|---|
### Chart
| Category | [O]_Posttranslational_modification,_protein_turnover,_chaperones |
|---|---|Number of the genes
Number of the genes
Genome size (Mbp)
Genome size (Mbp)

## Slide 5
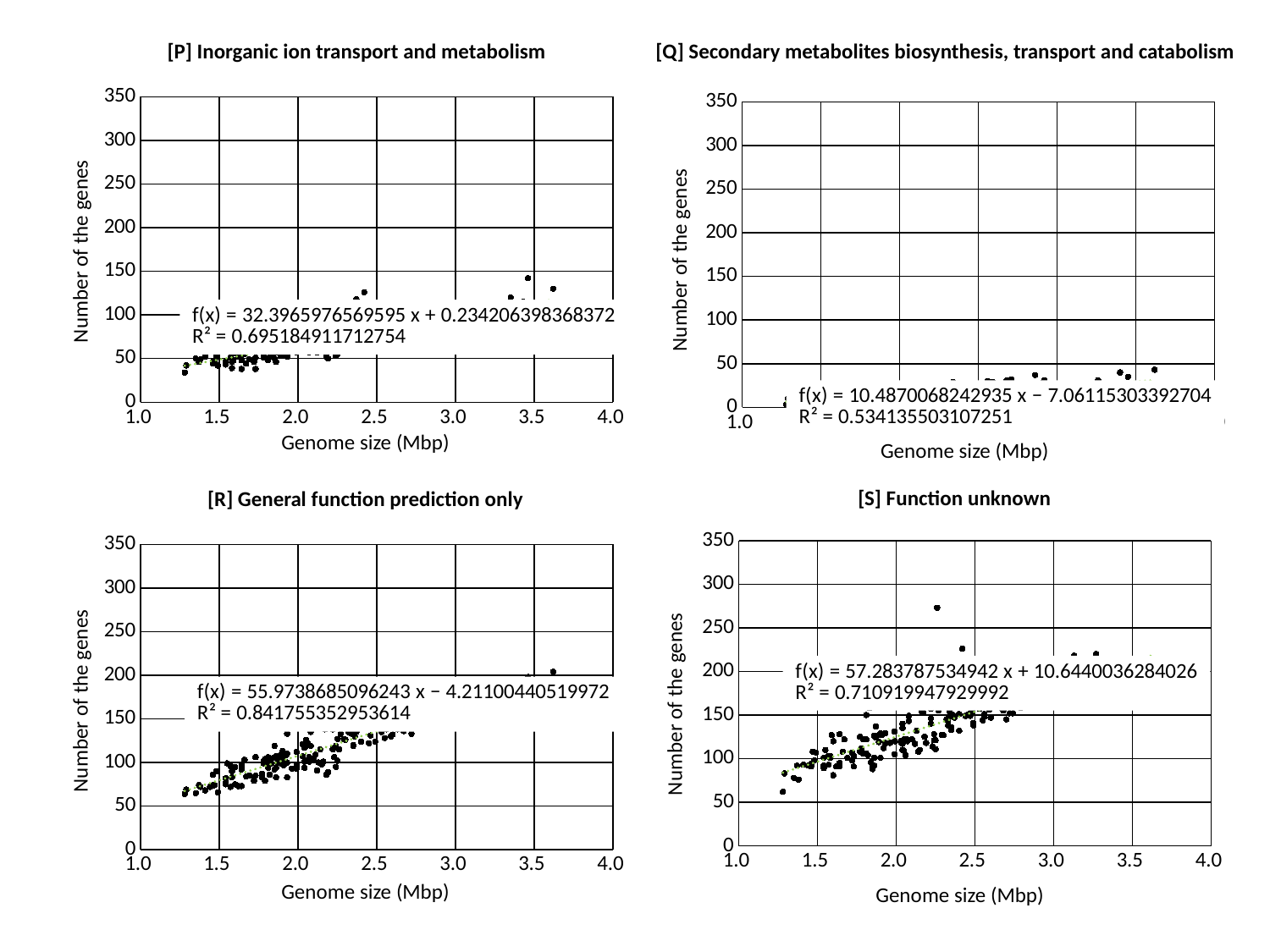

[P] Inorganic ion transport and metabolism
[Q] Secondary metabolites biosynthesis, transport and catabolism
### Chart
| Category | [P]_Inorganic_ion_transport_and_metabolism |
|---|---|
### Chart
| Category | [Q]_Secondary_metabolites_biosynthesis,_transport_and_catabolism |
|---|---|Number of the genes
Number of the genes
Genome size (Mbp)
Genome size (Mbp)
[S] Function unknown
[R] General function prediction only
### Chart
| Category | [S]_Function_unknown |
|---|---|
### Chart
| Category | [R]_General_function_prediction_only |
|---|---|Number of the genes
Number of the genes
Genome size (Mbp)
Genome size (Mbp)

## Slide 6
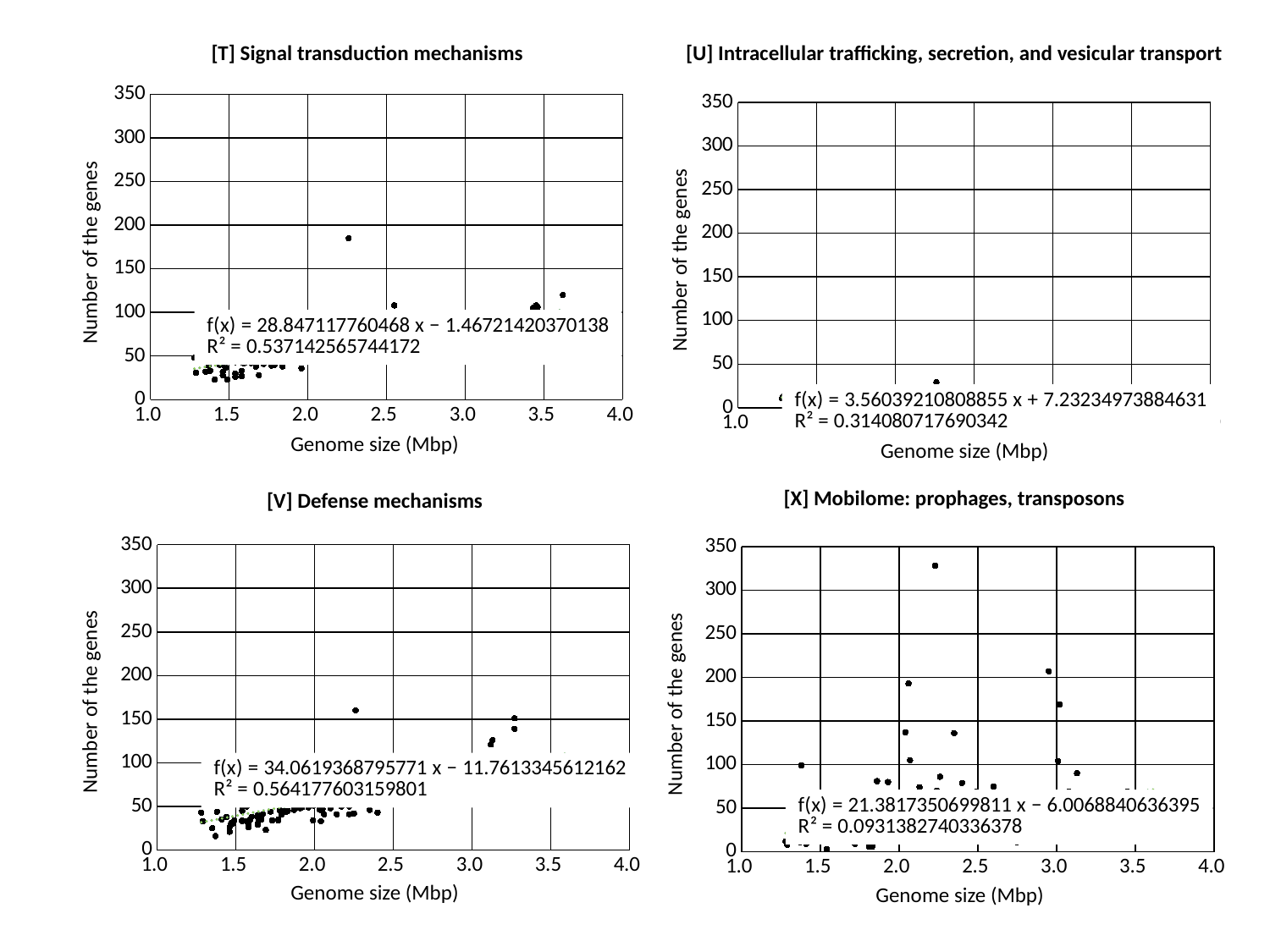

[T] Signal transduction mechanisms
[U] Intracellular trafficking, secretion, and vesicular transport
### Chart
| Category | [T]_Signal_transduction_mechanisms |
|---|---|
### Chart
| Category | [U]_Intracellular_trafficking,_secretion,_and_vesicular_transport |
|---|---|Number of the genes
Number of the genes
Genome size (Mbp)
Genome size (Mbp)
[X] Mobilome: prophages, transposons
[V] Defense mechanisms
### Chart
| Category | [V]_Defense_mechanisms |
|---|---|
### Chart
| Category | [X]_Mobilome:_prophages,_transposons |
|---|---|Number of the genes
Number of the genes
Genome size (Mbp)
Genome size (Mbp)
